# Laboratory Analysis of the Renal Function Changes Under Long-Term Exposure to Extremely Low Ambient Temperatures: Case Report

**DOI:** 10.1089/ther.2023.0086

**Published:** 2024-03-05

**Authors:** Aneta Teległów, Beata Skowron, Valerjan Romanovski

**Affiliations:** ^1^ Department of Health Promotion, Institute of Basic Sciences, University of Physical Education in Krakow, Krakow, Poland.; ^2^ Diagnostyka S.A., Kraków, Poland.; ^3^ Non-Governmental Organization and Associaton Oswajamy Zywioly, Kielce, Poland.

**Keywords:** renal function, extreme low temperature, laboratory analysis, case study

## Abstract

The study subject was a healthy, 47-year-old man, a low temperature Guinness World Record holder. He spent 50 days alone in Rovaniemi, Lapland, and functioned in the ambient temperature ranging from +2°C to −37°C. He did not use sources of heat, he did not eat warm meals or drink hot water, and did not dry his clothes. He slept in an igloo, on an ice cover of 20–30 cm. He spent 10 hours a day in a sleeping bag and for the remaining time he walked, skied, or rode a bicycle, and practiced swimming. The aim of the study was a laboratory assessment of renal capacity in a man exposed to long-term extremely low ambient temperatures. The study was approved by the Ethical Committee at the Regional Medical Chamber in Krakow, Poland (approval No.: 194/KBL/OIL/2019). Twice during the observation, urine and blood were collected and analyzed: before and after the prolonged exposure to extremely low ambient temperatures. Changes were seen in many blood and urine parameters, but in urine, they were more significant. In urine, decreased values of sodium (by 53.9%), potassium (by 22.6%), creatinine (by 65.5%), urea (by 61.3%), uric acid (by 58.4%), and protein (by 50%) were observed. Neutrophil gelatinase-associated lipocalin (NGAL) increased by 34%. Absence of calcium oxalate excretion was reported relative to the value before the exposure to cold. In blood, increased values of interleukin-6 (by 60%) and β-2-microglobulin (by 26.9%) were observed. Erythropoietin decreased by 22.4%. No changes were noted in estimated glomerular filtration rate. The study subject lost 10 kg in weight. On the basis of the results obtained during the observation, it can be determined that the probable cause of changes in the laboratory results of the subject was the diet used, and not a dysfunction of the excretory system. The body weight loss and activation of compensating mechanisms focused on saving vitally important diet components, caused by the insufficient diet, exclude the theory of a negative effect of exposure to extremely low temperatures on renal filtration function.

## Introduction

Evolutionary adaptation of humans to low-temperature climate conditions is still poorly understood. Regular exposure to the cold factor increases cold tolerance through numerous adaptive mechanisms (Teległów et al., 2022; Teległów et al., 2021; Teległów et al., 2016; Teległów et al., 2014). The human body adapts to low temperatures by means of thermoregulation. Of great importance are adaptations related to the blood vessel system (Teległów et al., 2022; Teległów et al., 2014). The body response to low temperature involves hormonal changes in the circulatory, nervous, and muscular systems (Knechtle et al., 2020). Previously, Teległów et al. (2022) evaluated the morphological and biochemical blood properties in a multiple Guinness World Record holder Valerjan Romanovski, who was exposed to extremely cold environment, with temperatures ranging from +2°C to −37°C, for 50 days in Rovaniemi (a city in northern Finland). They concluded that the subject's long-term exposure to extreme stress due to cold had no noticeable negative impact on the daily functioning. The stimulating factors were not only the extreme cold but also physical activity and methods of keeping warm (Teległów et al., 2022).

With that said, what is the mechanism for changes in kidney function under prolonged exposure to extremely low ambient temperatures?

The most important and routinely performed laboratory tests assessing the renal performance include measurement of creatinine and glomerular filtration rate. It is typically supplemented with the concentrations of final nitrogenous waste products—urea and uric acid (Traynor et al., 2006). However, it should be emphasized that renal performance affects considerably higher number of vital parameters, including, but not limited to, the count of red blood cells (RBC), hematocrit (HCT) level (Jelkmann, 2011), ion concentration (Hamm et al., 2015), or total protein level (Lamb et al., 2009), and even vitamin D (VitD) level (Holick, 2009). The complete image of the renal function is provided by the list of general and specialized urine testing with blood testing results, imaging and histopathological tests. A detailed analysis of the mentioned parameters enables the assessment of the excretory system and determination if its possible damage.

Renal function depends on numerous factors and efficacy of other systems. However, it largely depends on appropriate organism hydration (Danziger and Zeidel, 2015), status of blood vessels (Ruiz-Hurtado and Ruilope, 2018), supply of salt in the diet (Boero et al., 2002), body weight (Câmara et al., 2017), and drug use (Radi and Khan, 2019). Due to low number of scientific reports on the influence of external temperatures on the functions of the excretory system, in this article, we assess the impact of long-term exposure to extremely low temperatures on renal function.

The aim of the study was a laboratory assessment of renal capacity in multiple Guinness World Record holder Valerjan Romanovski, who was exposed to extremely low ambient temperatures, ranging from +2°C to −37°C, for 50 days in Rovaniemi (a city in northern Finland).

## Materials and Methods

### Study subject

The investigated subject was a multiple Guinness World Record holder for low temperatures living in Poland, Valerjan Romanovski, who was exposed to air temperatures ranging from +2°C to −37°C while staying in Rovaniemi at the mouth of the Ounasjoki River to Kemi during a research expedition (Teległów et al., 2022). The daily records of outdoor temperatures are presented in a separate publication (Teległów et al., 2022). The participant was healthy, with no comorbidities. His lifestyle and diet before the expedition were related to staying in cold temperatures and winter swimming to adapt the body to low temperatures.

The subject slept alone in an igloo situated on the river, on a 20–30 cm thick ice sheet. He separated himself from the ice with a piece of wood; he also used sheepskin, a sleeping pad, and bubble wrap. There were days lasting for 3 hours and nights of 21 hours during the expedition. The participant spent 10 hours/day in his sleeping bag. In the remaining time, in order not to freeze, he maintained physical activity, walking for 5–8 hours, ∼20 km/day, as well as skiing and cycling on the ice. He moved all the time. He wore goose down overalls, but only in temperatures lower than −25°C; in higher temperatures, he used survival multilayer clothing. His footwear was 2–3 sizes larger, which allowed him to put on a shoe after it had completely ossified; there was also a felt insole inside each shoe. The subject did not consume hot meals or dry his clothes. His only source of heat was the energy produced by his own body. During the expedition, heart rate was monitored with a Garmin Fenix X6 multisports watch (Finland) (Teległów et al., 2022).

During daytime, the man urinated 3–4 times/day. The frequency of urinating did not change during the expedition (3–4 times/day), whereas at night, the subject urinated 2 or 3 times.

The participant has provided his written consent for the publication of this article.

### Sampling and analysis of blood

Twice during the observation, blood was collected from the subject's ulnar vein: prior and after the prolonged exposure to extremely low ambient temperatures. The blood was collected at the Laboratory of Blood Physiology of the Academy of Physical Education in Krakow before the expedition—on November 11, 2020, and after return from the expedition—on February 2, 2021. The blood was collected to 2 test tubes (Becton Dickinson), with K2 EDTA anticoagulation agent to perform blood counts (RBC, HCT), and sample without anticoagulation agent, to perform further analyses at the Laboratory of Analytics and Clinical Biochemistry of the Institute of Oncology in Krakow.

Blood counts (RBC, HCT) were performed on the Advia 2120i analyzer (Siemens Healthineers). Erythropoietin (EPO), interleukin-6 (IL-6), and VitD were determined on the Alinity I immunochemistry analyzer (Abbott). The remaining parameters (sodium in plasma [NaP], potassium in plasma [KP], chlorides in plasma [ClP], inorganic phosphates in plasma [Pi], calcium in plasma [CaP], urea in plasma [UreaP], creatinine in plasma [CreaP], estimated glomerular filtration rate [eGFR], uric acid in plasma [UricP], total protein in plasma [ProteinP], albumin in plasma [AlbP], β-2-microglobulin) were determined on a Cobas 6000, model C501 biochemical analyzer (Roche). RBC and HCT concentrations were presented by Teległów et al. (2022) when assessing the morphological and biochemical blood properties in the investigated subject.

### Sampling and analysis of urine

Twice during the observation, in the morning, the subject collected fasting urine samples: before and after the prolonged exposure to extremely low ambient temperatures. General urine analysis (SG, pH, glucose, ketone bodies, urobilinogen, bilirubin, nitrites, color, and clarity) was performed on the Cobas u411 analyzer (Roche) with the use of Combur-10-M test strips (Roche). In addition, after centrifuging of the urine (5 minutes, 2500 rpm), a microscopic assessment of the sediment was performed (leukocytes, erythrocytes, mucus, bacteria, epithelium, minerals, and casts). Sodium in urine (NaU), potassium in urine (KU), chlorides in urine (ClU), urea in urine (UreaU), creatinine in urine (CreaU), uric acid in urine (UricU), and protein in urine (ProteinU) were determined on the biochemical Cobas 6000 analyzer, model C501 (Roche). Neutrophil gelatinase-associated lipocalin (NGAL) was determined on an Alinity I immunochemistry analyzer (Abbott).

### Calculation methods

Based on the obtained results of blood and urine testing, the following coefficients were calculated: UreaP/CreaP, CreaU/CreaP, UreaP/UreaU, renal failure index (RFI), fractional excretion of sodium (FENa), fractional excretion of urea (FEUrea), and fractional excretion of potassium (FEK). Results of the observed man prior the experiment were treated as control. Based on these results, the % value at which the result obtained after long-term exposure to extremely low ambient temperatures changed was calculated.

## Results

### Biochemical findings in blood

Minor changes between the results before and after exposure to extremely low ambient temperatures were observed in the renal function dependent blood counts indicators. The RBC was reduced by 4.01%, whereas HCT by 3.40% relative to the initial value. Level of electrolytes (KP, ClP) was maintained at a similar level both before and following the exposure to extremely low ambient temperatures, similar to CaP and UricP levels. Reduction of the level of analytes relative to the value before exposure to extreme cold was observed for UreaP, ProteinP, AlbP, and EPO. Increased concentration of analytes relative to the value before exposure to extreme cold was observed for CreaP, VitD, IL-6, and β-2-microglobulin. No changes were observed for NaP and eGFR (Table 1).

**Table 1.** Results of the Subject Before and After Long-Term Exposure to Extremely Low Ambient Temperatures

Analyzed parameter (unit of measure)	Result before exposure to extreme cold	Result after exposure to extreme cold	Change compared to the result before (%, no change, change was observed)
Body mass			
Weight (kg)	85.5	76.3	10.8
Blood			
RBC (xlO^12^/L)	4.74	4.55	−4.01
HCT (%)	44.1	42.6	−3.40
NaP (mmol/L)	139	139	No change
KP (mmol/L)	4.49	4.52	+0.67
C1P (mmol/L)	100.9	102.2	+1.29
Pi (mmol/L)	0.86	1.07	+24.42
CaP (mmol/L)	2.44	2.39	−2.05
UreaP (mmol/L)	3.18	2.71	−14.78
CreaP (μmol/L)	68.8	73.6	+6.98
eGFR (mL/min)	>90	>90	No change
UricP (μmol/L)	247.3	251.6	+1.74
ProteinP (g/L)	76.8	71.4	−7.03
VitD (ng/mL)	18.7	22.4	+19.79
AlbP (g/L)	48.2	44.2	−8.30
EPO (mIU/mL)	13.4	10.4	−22.39
IL-6 (pg/mL)	1.5	2.4	+60
β-2-microglobulin (mg/L)	1.56	1.98	+26.92
Urine			
NaU (mmol/L)	175	76	−53.94
KU (mmol/L)	50.4	39	−22 62
C1U (mmol/L)	163.3	69.9	−57.20
UreaU (mmol/L)	421.1	163.1	−61.27
CreaU (μmol/L)	24231.5	8354.4	−65.52
UricU (μmol/L)	2911	1210.3	−58.42
ProteinU (mg/L)	100	50	−50
NGAL (ng/mL)	10	13.4	+34
SG (g/mL)	1.025	1.020	−0.49
pH	5	5	No change
Glucose	Negative	Negative	No change
Ketone bodies	Negative	Negative	No change
Urobilinogen	Negative	Negative	No change
Bilirubin	Negative	Negative	No change
Nitrites	Negative	Negative	No change
Color	Light yellow	Light yellow	No change
Clarity	Clear	Clear	No change
Leukocytes^a^	0–2	0–2	No change
Erythrocytes^a^	0–2	0–2	No change
Mucus^a^	Negative	Negative	No change
Bacteria^a^	Negative	Negative	No change
Epithelium^a^	Negative	Negative	No change
Minerals^a^	Calcium oxalate	Negative	Change was observed
Casts^a^	Negative	Negative	No change
Calculation parameters			
UreaP/CreaP	24.54	19.55	−20.33
CreaU/CreaP	352.20	113.51	−67.77
UreaP/UreaU	132.42	60.18	−54.55
RFI (mmol/L)	0.47	0.67	+42.55
FENa (%)	0.34	0.48	+42.43
FEUrea (%)	37.59	49.56	+31.84
FEK (%)	3.18	7.6	+138.99

^a^ In the field of view.

AlbP, albumin in plasma; CaP, calcium in plasma; CreaP, creatinine in plasma; CreaU, creatinine in urine; eGFR, estimated glomerular filtration rate; EPO, erythropoietin; FEK, fractional excretion of potassium; FENa, fractional excretion of sodium; FEUrea, fractional excretion of urea; HCT, hematocrit; IL-6, interleukin-6; KP, potassium in plasma; KU, potassium in urine; NaU, sodium in urine; NGAL, neutrophil gelatinase-associated lipocalin; NaP, sodium in plasma; Pi, inorganic phosphates in plasma; ProteinU, protein in urine; RBC, red blood cells; RFI, renal failure index; UreaP, urea in plasma; UreaU, urea in urine; UricP, uric acid in plasma; UricU, uric acid in urine; VitD, vitamin D.

### Biochemical findings in urine

Among the urine testing results following the exposure to extremely low ambient temperatures, a slight decrease of SG was observed and absence of calcium oxalate excretion relative to the value before the exposure were determined. Reduced level of analytes relative to the value before exposure to extreme cold was observed for NaU, KU, ClU, UreaU, CreaU, UricU, ProteinU, UreaP/CreaP, CreaU/CreaP, and UreaP/UreaU. Increased concentration of analytes relative to the initial values before the exposure to extreme cold were observed for NGAL, RFI, FENa, FEUrea, and FEK. No changes were found for pH, glucose, ketoacids, urobilinogen, bilirubin, nitrates, color, clarity, leukocytes, erythrocytes, mucus, bacteria, epithelia, and cellular casts (Table 1).

## Discussion

The interstitial structure of kidneys consists of type I and II cells, macrophages and dendritic cells. These cells are responsible for the synthesis and decomposition of extracellular matrix, as well as for the production of EPO and prostaglandins. They support the structure of nephron and constitute the route of electrolyte transport (Duffield, 2010; Okusa and Li, 2012). Hypoxia of interstitial cells due to reduced flow of blood through the kidney is the main cause of the physiological increase of EPO production, which in turn stimulates the hematopoietic system to increase the production of RBC (Shih et al., 2018).

However, in the analyzed participant, the EPO production decreased by 22.39% with concomitant reduction of RBC and HCT. These results exclude the theory of the organ hypoxia, caused by the possible shrinking of blood vessels produced by exposure to extremely low temperatures. Vasoconstriction is a physiological response to low temperature, protecting the body from heat loss (Teległów et al., 2022). The loss of functional renal mass has been mentioned as the main cause of the EPO drop, which is most often caused by chronic or acute kidney disease (de Seigneux et al., 2012; Nakhoul and Simon, 2016). Analysis of the subject results (UreaP, CreaP eGFR, and UricP) in the mentioned causes of EPO can be excluded because the concentration of UreaP decreased, CreaP increased slightly, and no changes in terms of eGFR and UricP could be observed. Thus, it can be presumed that the reduction of EPO production (a peptide hormone) could be linked to weight loss of the subject.

The EPO receptor expression was also observed in cells other than erythroidal cells: nervous, endothelial, and musculoskeletal cells (Suresh et al., 2019). The weight loss, resulting probably from the loss of both fat and muscle tissue, could cause a reduction in the amount of receptors for EPO, which in turn would lead to be proportional to the number of receptors to reduce the production of hormone itself. As a consequence of this change, the RBC production decreased and the HCT value was reduced. The reduced hormone production could also result from a reduced number of substrates (amino acids) necessary for the synthesis of the peptide hormone. This theory is supported by Rose (2019), who maintains that peptide hormones play a major role in mediating the metabolic response to changes in protein intake.

Based on the subject's weight loss, it can also be concluded that the subject's diet did not provide the body with a sufficient amount of calories, and the energy consumption used to maintain normothermic exceeded its supply. This relationship can be clearly seen in the ProteinP results, the concentration of which was reduced by 7.03%, and AlbP whose concentration was reduced by o 8.30%. The frequent cause of such reductions in patients is proteinuria, resulting from an impaired kidney function. Proteinuria is defined as the pathological presence of the protein in the urine in the amount above 150 mg/24 hours. It can be a consequence of prerenal (Cuenca-Sánchez et al., 2015), renal (Couser, 2017) disturbance and urinary tract dysfunction (Lee, 2019).

Proteinuria characteristic of kidney diseases results from two pathological mechanisms: damage to the kidney glomeruli and their cells—podocytes, or/and damage to tubules (Skowron et al., 2019). ProteinU results, the concentration of which decreased by 50% throughout observation, preclude proteinuria as the cause of ProteinP and AlbP reduction in the subject. Such a tendency proves that extreme conditions forced the body to launch a compensation mechanism involving protein saving, due to its insufficient supply in the diet.

Lack of sufficient dietary protein intake is also shown for the analysis of UreaU CreaU, UricU parameters, the concentration of which in the urine relative to the period before exposure to extremely low temperature decreased by: 61.27%, 65.52%, and 58.42%, respectively. UreaU, CreaU, and UricU are protein/nitrogen metabolism products, whose excretion level evaluation is used in the diagnosis of renal filter efficiency. Renal disorder is indicated by increased UreaP, CreaP, and UricP concentration above the reference values with the concomitant reduction of UreaU CreaU, and UricU excretion caused by the impairment of the filtration function, resulting in reduced eGFR. Reduced UricU excretion with the increase of UricP concentration is the cause of crystallization of uric acid within the renal channel and an inflammatory process in kidneys (Kim et al., 2000; Nakagawa et al., 2006).

In addition, the increased concentration of uric acid by inhibiting nitrogen monoxide synthesis, reduction in the production of nitric oxide and stimulation of the renin—angiotensin—aldosterone system leads to renal vasoconstriction and impairment of kidney function (Ejaz et al., 2007; Khosla et al., 2005). In the observed subject, the tendencies of changes in the concentrations of the mentioned indicators exclude the impairment of the kidney function, while proving the protein-saving mechanism exhibited by the organism, which mainly confirms the almost 15% decrease in the concentration of UreaP—the final product of protein transformation, while decreasing the concentration of UreaU and the absence of changes in EGFR.

A similar compensatory mechanism, consisting in saving important substances for the body, was observed in the investigated subject of a water and electrolyte metabolism. Analysis of the ion concentration in the serum (NaP, KP, and ClP) showed no significant deviations relative to the ion concentrations observed before the period of exposure to extremely low ambient temperatures. A completely different situation concerns the urine analysis. Concentrations of NaU, KU, and ClU decreased by: 53.94%, 22.62%, and 57.20%, respectively, relative to concentrations before the exposure to low temperatures. Reduced excretion of ions probably resulted from the reduced supply of electrolytes in the diet and the necessity of their maximum usage by the body. Electrolyte disorders in the form of hyperkalemia and hyponatremia are often diagnosed when renal disorder is identified (Gerigk et al., 1995; Gil-Ruiz et al., 2012; Samimagham et al., 2011; Watanabe, 2004).

Hyponatremia is mainly the result of overhydration associated with the impairment of diuresis—anuria or oliguria, while hyperkalemia develops as a consequence of disturbed potassium excretion through damaged tubuli (Pandey et al., 2016). In the study by Hsieh et al. (2011), it was calculated that in the course of chronic renal disease every GFR decrease by 10 mL/min generates increase of serum potassium concentration by 0.117 mmol/L. No change in eGFR and electrolyte disorders could be observed in the present analysis. The intensified back absorption of ions by kidneys, which resulted in their reduced excretion probably resulted from the decreased supply of electrolytes in the diet, and the amount of water supplied both contained in the food and in a liquid form, which was markedly lower compared to the period from before exposure to extremely low temperatures.

Microscopic assessment of the subject's urine also shows a strong impact of the diet on the results obtained after the exposure period. Presence of minerals—calcium oxalate—was observed in urine testing before the expedition. After the return the minerals were not present in the microscopic image of urine. Oxalate calcium in the urine occur in patients whose diet is rich in spinach, sorrel, rhubarb, chard, dried figs, chocolate, cocoa, strong tea, coffee, meat and fish cans, pickles, concentrates of soups and sauces, hot spices, and spices containing sodium glutamate. The elimination of these products leads to a reduction or complete elimination of excretion of calcium oxalate with urine (Mitchell et al., 2019). The diet used by the subject during the expedition contained no products that would maintain oxalate levels, in connection with the above, the only explanation of the observed change is the diet eliminating these products.

Due to the considerable changes of the analyzed indicator parameters observed primarily for urine of the subject, NGAL and β-2-microglobulin determinations were also conducted, which are known as renal impairment markers. Following literature reports, NGAL concentration in urine increases already 2 hours following an injury, caused mainly by the organ's hypoxia, related to abnormal flow of blood through kidneys (Lisowska-Myjak, 2010). On the other hand, increased concentration of creatinine and other metabolites is observed only when a significant loss of active nephrons occurs, and GFR drops by half (Traynor et al., 2006).

In the observed participant, the NGAL increased by 34% relative to the period before the expedition. However, despite the considerable increase in concentration, the NGAL value remained in the reference value range (0–131.7 ng/mL) for the analyzed indicator, which in turn precludes damage of the filtration function of the organ. Because apart from damaged nephron, NGAL is also produced in many other extrarenal tissues (e.g., prostate, uterus, trachea, stomach, liver, lungs, and large intestine) and the growth of its concentration is further caused by the immune cells—neutrophils and macrophages (Dobrek and Thor, 2016), the observed increase in NGAL concentration could result from its increased, probably extrarenal production (e.g., prostate stimulated by cycling).

A considerable increase (26.92%) compared with period before the expedition was also observed for β-2-microglobulin, a parameter used, among others, for differentiation of renal dysfunction causes (Li et al., 2016). As a result of the filtration renal functional impairment and the progressing organ damage, the β-2-microglobulin concentration increases in the serum. However, in this investigation, despite the considerable elevation of the analyzed marker, its value was still within the reference range for the analyzed indicator (0.8–2.2 mg/L), which as for NGAL, excludes the theory of renal damage caused by prolonged exposure to extremely low ambient temperatures.

The role of IL-6 pro-inflammatory cytokine is to initiate acute-phase protein synthesis in hepatocytes. According to literature data, in the context of the urinary system, the IL-6 concentration elevation is observed, among others in patients with increasing severity of renal damage (AKI marker), it also correlates with the severity of proteinuria (Magno et al., 2019). In the analyzed participant, a 60% increase in IL-6 concentration was observed compared to the value from before the expedition. However, similar as for β-2-microglobulin and NGAL, the increased concentration did not exceed above the range of reference values of an adult person (0–7 pg/mL), which again precludes the renal functional impairment.

It is difficult to refer to Pi, CaP, and VitD values due to the supplementation used by the subject. Conditions experienced by the subject (day and night length, latitude, season, and sunlight angle) excluded the endogenous synthesis of VitD. The increase in VitD concentration in relation to the result from before the expedition and changes in Pi and CaP concentrations can be only explained by the influence of exogenous supplementation of VitD on the subject's calcium-phosphate metabolism.

The multitude of results enabled calculation of numerous coefficients: UreaP/CreaP, CreaU/CreaP, UreaP/UreaU, RFI, FENa, FEUrea, and FEK. Considering their use mainly in the recognition of clinical form of acute kidney damage (AKI), they do not bear a significant importance in the present analysis due to the lack of the above diagnosis for the observed subject according to the applicable criteria (*Ad hoc* working group of ERBP et al., 2012). These results only constitute an interesting supplement to the obtained data.

With regards to pain experienced by the observed subject during the expedition, no symptom in the form of back pain was found, which can characterize patients with certain kidney diseases (causing increased kidney volume).

## Summary

Based on the results obtained during observation, it can be determined that the probable cause of changes in the laboratory results of the subject was the diet used, and not the dysfunction of the excretory system. The body weight loss and activation of compensating mechanisms focused on saving vitally important diet components, caused by the insufficient diet, exclude the theory of the negative effect of exposure to extremely low temperatures for renal filtration functions. However, it should be added that a precise and reliable determination of the causes of the observed changes, would in theory require repeating the observation, while choosing a properly balanced diet, covering the energy demand for a person exposed to long-term extremely low temperatures.

## Data Availability

The original contributions presented in the study are included in the article/supplementary material, and further inquiries can be directed to the corresponding author/s.
